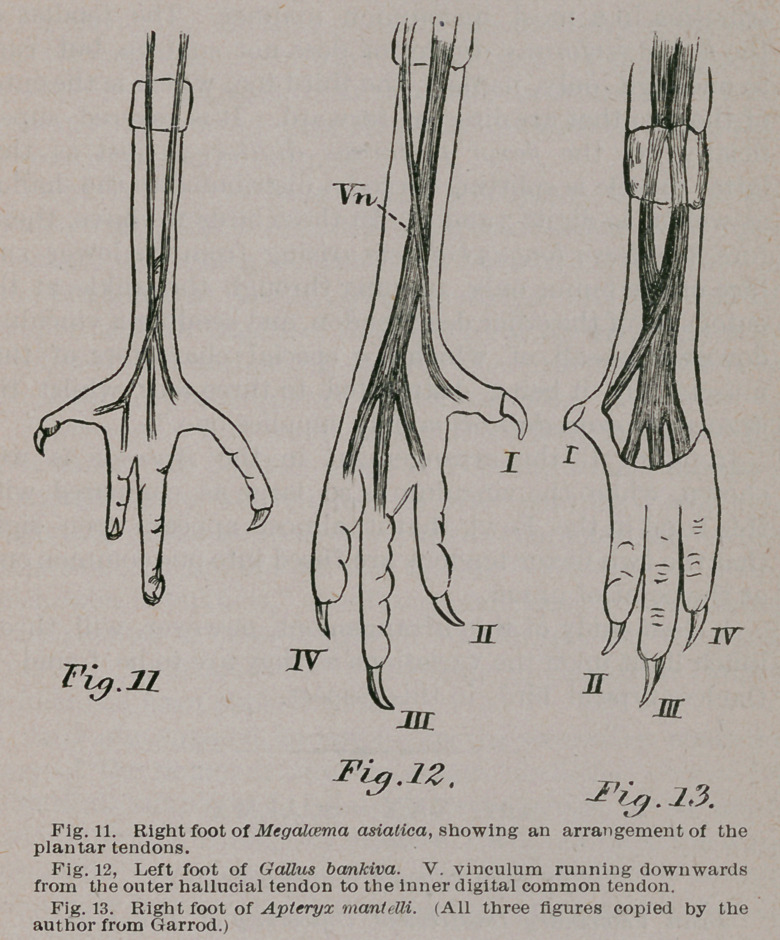# A Review of the Muscles Used in the Classification of Birds

**Published:** 1887-10

**Authors:** R. W. Shufeldt

**Affiliations:** U. S. A.; Captain Medical Corps U. S. Army


### Art. XXIX. A REVIEW OF THE MUSCLES USED IN THE CLASSIFICATION OF BIRDS.

BY R. W. SHUFELDT, M.D., C. M. Z. S.

*Captain Medical Corps U. S. Army.*

When Garrod came to demonstrate that there were certain muscles, and groups of muscles in Birds that could be used in them to classificatory ends, he made the majority of his dissections upon Old World forms, and it was the exception when any of our United States bird-types came beneath his skillful scalpel for this purpose. Then, again, his investigations were for the most part published in the Proceedings of the Zoological Society of London where they are not as easily accessible to all American students as they might be ; to be sure, Garrod's papers were, after his death, gathered together in a handsome volume of "Scientific Memoirs," but even this work is now already too scarce, and but few copies of it have found their way to this country. In this latter work, too, his excellent labors in this line are scattered through a large volume, and we find no single chapter with appropriate figures especially devoted to all the muscles which up to the present time have proved to be so useful, as one set of characters at least, in determining the affinities of avian groups. Moreover, the present writer has recently added to the list another muscle which he has reason to believe will prove of value in certain orders of birds, both for classification and as an indication with respect to affinity, among others, of these interesting types of vertebrates. (See *Science*, No. 229, p. 623, and No. 234, p. 57). In view of these facts, I have reason to believe that such an illustrated chapter as is alluded to above will prove helpful to many anatomical students upon this side of the water, and to this end the present article will be devoted.

Garrod dealt chiefly with a certain group of muscles which pertain to the pectoral limb in birds; with others that occurred in their thighs; with the obturator internus; with the plantar tendons; and finally, with the musculature of the lower larynx.

In passing I may say here that I am fully convinced that when the myology of birds comes to be still better known, and generally worked out, we will find many other muscles and arrangements of their tendons in various parts

Fig. 1.

Fig. 1.—View, from the outer side, of the muscles of the patagium of the left wing of a passerine bird. Troupial *Icterus vulgaris* (after Garrod).

of the body, as in the pinion, in the leg, and elsewhere, which also will come into use in the taxonomy of the class. Here, I will have but little or nothing to say in reference to the muscles of the larynx, as we must believe that our knowledge of them in birds has not as yet arrived to that state of perfection where we can efficiently employ them in special classifications. It will be to our purpose to dwell quite fully, however, upon the classificatory muscles of the pectoral and pelvic limbs; of such use as the form of the area of origin of the obturator internus has proved to be; and lastly, a few words with respect to the variations exhibited on the part of the plantar tendons in certain groups of birds.

Let us first, then, turn our attention to the muscles of the *pectoral limb*, and up to the present time the *five* following named ones have been found useful as characters in the classification of the class, or have proved of assistance, when

Fig. 2.

Fig. 2.—Same view and corresponding parts of the same wing of a passerine bird, Purple Martin, *Progne subis*, (by the present writer). *tp. l.*, tensor patagii longus; *tp. b.*, tensor patagii brevis; *dt. p.*, dermo-tensor patagii; *e. m. r. l.*, extensor metacarpi radialis longus; *t.*, triceps; *b.*, biceps; *S. R.*, secondary remiges (fig. 2 nearly × 2).

taken in connection with other structures, in determining affinities.

1. *The tensor patagii longus.*
2. *The tensor patagii brevis.*
3. *The dermo-tensor patagii.*
4. *The bicipital slip to the patagium.*
5. *The expansor secundariorum.*

Garrod found all but No. 3 of these present in one bird

(*Chauna derbiana*), but I have yet to know of one that possesses them all.

Some birds have the first and second with all the others absent; many possess the first three mentioned in the list with the last two absent; and so on. Then, again, they vary, as I say, in their form; their modes of origin and insertion; and in their special modifications. And while all this is very fortunate for the purposes of classification, it will be impossible in an article of the present limits to think of entering upon such matters in their detail. My aim will have been accomplished by simply giving a good description and figure of each of these muscles, and then the student may study their modifications in other bird-forms to suit himself. Should his library contain the Collected Memoirs of Garrod, and his successor, W. A. Forbes, to the prosectorship of the Zoological Society of London, so much the better, for then indeed will he have capital assistance at hand. Forbes, for instance, found some wonderfully interesting and complicated conditions of these muscles in certain sea-fowl. (*Tubinares.*)

Any good passerine bird, as an oriole, for example, has in the musculature of its wing the first three mentioned in the list; and when I say any passerine bird I mean in so far as I have yet examined them. Having secured a good specimen of some such bird, pluck it perfectly clean, being careful, in removing the feathers of the wings, that you do not tear the skin. (Fig. 3). Now with a small, sharp dissecting scalpel make an incision just through the integuments and no more, along the line which I have indicated by the letters *inc.* in figure 3, and then carefully and completely reflect this skin-flap in both directions until all the muscles of the arm and forearm are exposed, even to the tendon which extends from the shoulder to the wrist (*tp. l.*), in the free margin of the duplicature of the skin, here called the *patagium*, in which these muscles are found.

1. The *tensor patagii longus* (Fig. 3, and elsewhere *tp. l.*) Among the *Passeres* this muscle is found to consist of a small fusiform, carneous portion, and a long delicate tendon. Arising as a short, flat fasciculus of fibres, from the head of the corresponding coracoid bone, being superficial to the carneous portion of the *tensor patagii brevis* with which it is connected by fascia, and still more intimately

Fig. 3.—The plucked body of a medium sized passerine bird, with its pterylosis not shown, but with the *patagial muscles* of the arm in black, and supposed to be seen through the skin ; *dt. p.*, *dermo-tensor patagii; tp. l.*, *tensor patagii longus* ; and *tp. b.*, the *tensor patagii brevis* ; *inc.* the line of incision to be made in order to expose them for examination.

so with the adjacent *pectoralis major* muscle ; this muscle soon terminates in a tendon, which latter supporting the free margin of the patagial membrane, runs directly in it to the carpal joint, where it becomes attached, often sending a few tendinous fibres over it to be inserted into the pollex phalanx. By following the directions given above, this muscle and its tendon can be easily brought into view in the wing of any passerine bird, as well as in the majority of others.

2. The *tensor patagii brevis* is a muscle that has been so well and clearly described by Garrod (Coll. Sci. Mem., p 356) that I will reproduce his original description here. He says: "In the triangular patagium of the wing of the bird the tendons of two muscles are to be found. One is that of the *tensor patagii longus*, which forms the supporting cord of the free margin of the membrane itself. The second is that of the *tensor patagii brevis*, which courses parallel with the humerus, not distant from that bone, to the muscles and fasciæ of the forearm (See Figs. 1–3 and 5–6 of the present paper, *tp. b.*) In the Ramphastinæ, Capitoninæ, and Picinæ, where this muscle is less complicated than in any other birds, it arises, as is generally the case, from the apex of the upper of the two processes at the scapular extremity of the furcula, as well as by a small special slip from the superficial fibres of the *pectoralis major* muscle, which differentiates itself off from the main muscle near the upper part of its inserted extremity. The comparatively insignificant triangular or compound fleshy belly thus formed, with its apex directed towards the elbow, terminatesin a cylindricaltendon, which, included between the layers of the fibro-cutaneous patagium, takes a straight course to its insertion into the axially-running tendon of the origin of the *extensor metacarpi radialis longus* of Schopss [*e. m. r. l.* in the figures here given], at a short distance from the tubercle on the humerus whence the muscle springs."

"As a result of this disposition, when the fore-arm is half-flexed, the tendon of the *tensor patagii brevis* is seen to enter the substance of the fibrous origin of the *extensor metacarpi radialis longus*, and at right angles. This arrangement is indicated in plate 21 [of his work], and is characteristic of the Picariæ, as defined by myself to include the three sub-families above referred to, and them only."

"Among the Passeres a slight, but easily recognizable difference in the manner of insertion of the muscle maintains. The similarly single cylindroid tendon runs from the muscular belly, which has its origin at the shoulder, as above described, to the upper margin of the *extensor metacarpi radialis longus* muscle, at an exactly similar spot: it does not, however, simply blend with the fibrous origin of that muscle: it becomes attached to it at the spot indicated, and then (again considering the forearm as half bent upon the humerus) runs back *independently* to be attached to the base of the tubercle of origin of the *extensor met. rad. longus*, slightly below that muscle's springing-point. As a consequence of this arrangement there are two tendons to be seen running to one spot (that on the upper margin of the *extensor met. rad. longus*, where the tendon of the *tensor* meets it) from two points, one the apex of the tubercle on the humerus above referred to, and the other, the depression at its base. These tendons therefore converge as they leave the elbow, having at first an appreciable interval between them, which is gradually diminished as they approach, although they remain quite free from one another, that of the *tensor* being superficial."

As I have already stated above, the insertion of this muscle is vastly different from that in other orders of birds, and some differences even occur among the passeres themselves, especially when we come to examine the more aberrant types of this vast group.

3. The *dermo-tensor patagii* (See figures 2 and 3 *dt. p.*) It is but a few weeks ago since I originally described this muscle (*Science*, No. 229, pp. 623,624, and 234, p. 57),and as I have not further investigated it since then, my remarks upon it can very appropriately be reproduced here. Of it, I said substantially that among my manuscripts in the hands of publishers, I have some very extensive work upon the myology of birds, illustrated by nearly a hundred original drawings; and, as many of my friends are aware, I have been engaged for a number of months past upon my second contribution to the anatomy of the Macrochires, a work now drawing towards completion. Quite recently, while investigating the muscular system of the Hirundinidae, in this latter connection, I discovered, in the course of my dissections, a muscle for which at this moment I recall no published description, and one the importance of which Garrod, even if he knew of its existence, certainly overlooked. When present, its chief carneous portion occurs in the free marginal fold of that triangular duplicature of the common integuments found between the root of the neck and the tip of the shoulder in birds. It first came to my notice in a specimen of *Progne subis*, whereupon I at once dissected a number of other individuals of the same species; and I found it equally well developed in all of them.

This muscle, in part, is a dermal muscle, and arises from the integuments on the anterior aspect of the neck at about its lower third; at its origin its fibres spread out fan fashion, their terminal fibres meeting those of the muscle of the opposite side in the median line. Here it is quite adherent to the skin, but its fibres rapidly converge as they pass in the direction of the shoulder-joint, opposite which region they gradually free themselves from the skin to form a small fusiform muscle, which, ending in a delicate tendon, runs along within the free marginal fold of the patagium of the wing, in common with the tendon of the tensor patagii longus, to blend with it just before arriving at the carpal joint.

Garrod chose the wing of *Icterus vulgaris* to illustrate the arrangement of the patagial muscles in the Passeres, and in his figure of it, a tendinal slip is shown cut short, of which he says nothing, but which evidently belongs to this muscle. Nowhere else is this shown or alluded to in his work.*

I propose to call this muscle the 'dermo-tensor patagii,' it being partially connected with the integumentary system of muscles in the birds wherein I have thus far found it.

Upon dissection, I find it present in each and all of the other United States Hirundinidae; in all true passerine birds, including *Ampelis;* but absent in the Caprimulgi, in the Trochili, in the Cypseli, and, if we may judge for all the typical Passeres mesomyodi from the condition in *Tyrannus tyrannus*, it is also wholly absent in them. Further than this, I have not investigated the matter, but it will be

*Plate 21, fig. 2, of his Coll., Sci. Mem., and Fig. 1, of the present paper.

highly interesting, to say nothing of its importance, to look up the subject for other groups of birds. Its importance at once becomes evident by finding it in such a form as *Ampelis*, showing by this character, at least, the passerine affinities of this bird over its clamatorial ones, which latter have been more than once suspected, at different times, as predominating in its organization.

4. The *bicipital slip to the patagium*, (figs. 5 and 6 *B. slip*). This is a fleshy fasciculus of muscle that is differentiated off from the anterior surface of the *biceps*, and passing between the cutaneous folds of the patagium becomes inserted into the tendon of the *tensor patagii longus* at about the middle of its course.

Garrod states that "the presence or absence of this muscular fasciculus is a very constant character among closely allied birds." He not only found it in the Caprimulgi, but also in Plovers, Cranes, Gulls, Auks and some few other groups. In figure 6, I present its appearance as it occurs in our Mourning Dove, a bird I especially dissected to show it as an illustration in the present connection.

Professor T. Jeffery Parker describes this muscle for the Common Pigeon (*Zootomy p.* 251) as the *tensor patagii accessorius*, and says "its anterior border is connected by fascia with the tendon of the tensor longus, and from its posterior border a long stout tendon is given off which passes outwards, soon becoming parallel to the tendon of the tensor longus, and having a common insertion with it."

If this last tendon be present in our wild pigeons, it is very feebly developed and consequently easily overlooked. I did not detect it in the Dove above alluded to, and must believe it was absent in that particular specimen. Whereever I examined it, it has invariably agreed with Garrod's description of it.

5. The *expansor secundariorum* (Fig. 5, *Exp. Sec.*), although of insignificant size, is a muscle that has proved of no little value as a classificatory one. Garrod spoke of it as the *Ciconine* character, as it was so well developed in the storks. It occurs in quite a large number of groups of

Fig. 7. Outer aspect of the right pelvic limb of the "Road Runner" (*Geococcyx californianus*) showing the superficial layer of muscles, and the first view of the *semimembranosus* and *semi-tendinosus*, two muscles used in classification. Life size by the author from his own dissections.

birds, as the *Gallinæ*; the Ducks, Geese, and Swans; the

Rails, Plovers and many others. While "in the majority of the Gallinaceous birds the *expansor secundariorum*, with the normal origin from the secondary quills, has a different method of insertion, which has led Mons. A. Milne-Edwards to describe the muscle in the Common Fowl as a part of the *coraco brachialis* (*brevis*) in his superb work on fossil birds" (Garrod).

Professor Sutton alludes to this muscle in the following interesting way. He says, "every student of human anatomy must have experienced a certain amount of curiosity when he dissected for the first time the plantaris muscle; this strange structure sinks into insignificance when compared with the celebrated ambiens of the bird's leg, or the tendon of the femoro-caudal in the lacertilia. Of all strange muscles, the one known as the *expansor secundariorum* (Garrod) in the bird's wing, stands pre-eminent. It is a small triangular muscle, arising from the quills of the last few secondary remiges at the elbow. Its remarkably long and slender tendon, which frequently traverses a fibrous pulley on the axillary margin of the *teres* muscle, runs up the arm side by side with the axillary vessels and nerves, to be inserted in the thorax into the middle of a tendon, which runs from the inner side of the middle of the scapular element of the scapulo-coracoid articulation, to near the thoracic border of the sterno-coracoid articulation, at right angles to it when the fore-limb is extended.

In the ducks and geese, among the Anseres, the tendons under consideration, when they enter the thorax, run towards one another and join (after having expanded out), in the middle line in front of the œsophagus, and behind the trachea.

My investigations into the morphology of this tendon induce me to believe that it is the representative in the bird's wing of the coraco-brachialis longus of mammals, and the long brachial ligament of man." (*Ligaments, their nature and morphology, p.* 33).

This will prove a very interesting muscle indeed to search for in the various forms of bird life in our own United States avifauna.

### OF THE MUSCLES IN THE THIGH OF BIRDS.

There are *five* muscles in the thigh which have proved to be more or less useful in the classification of Birds. These muscles are the following, and four of them I have designated by the letters which were used by Garrod in his myological formulæ.

6. *The ambiens,*
7. *The femoro-caudal,* . . A
8. *The accessory femoro-caudal,* . . . B
9. *The semitendinosus,* . . . X
10. *The accessory semitendinosus,* . . . Y

We know of no bird in which all five of these muscles are absent, or even of one which lacks the last four in the list.

According to Garrod, "when these four muscles are present in a bird the formula, AB. XY expresses the fact; when any one is absent, that such is the case is indicated by the omission of the letter representing it. Thus the formula A. XY indicates that the accessory femoro-caudal muscle only is absent; AB. X that the accessory semitendinosus is missing; A.X that the femoro-caudal and semitendinosus only are te be found; and A that the femoro caudal alone is present."

This eminent anatomist applied these myological formulæ to a classification of the entire group of existing birds, and fully discussed the matter in his work in the most masterly manner in so doing, but it will be impossible to enter upon any such field here. In my own opinion, however, I am inclined to believe that Garrod's classification stands in need of a very thorough overhauling in many of its aspects; by this I mean that in a vast number of cases we are not in possession of the requisite knowledge of the *entire structure* of certain forms as to warrant one retaining them where Garrod has placed them. In other words, these myological formulæ, as time goes by, and our knowledge of avian morphology widens, will surely prove very useful in taxonomy, *but* they can only be employed with safety when taken, as *one set of characters*, in connection with *all* the others that

Fig. 8. Pelvic limb, right side of *G. californianus*, same specimen as is shown in figure 7. The superficial layer of muscles have here been removed, and those found beneath them brought more fully into view. Life size by the author from his own dissections.

the organization of any particular bird-form presents us with, and by no means are we to rely upon them alone, or even when a few other sets of structural characters seem to indicate a bird's affinity.

To illustrate my point, let us turn for a moment to the Swifts and Humming Birds; here we have two groups which for years past have been associated together as allied forms by systematists, and Garrod, too, seemed to believe in their affinity. Why? Because the formula for the thigh muscles in each case was found to be A; the sternum had in each case an unnotched posterior border; and neither swifts nor hummers possess intestinal cœca. Yes, this all may be so, but *all* the rest of the organization of these birds is as widely different as one can well imagine, and consequently they belong to very different orders of birds. This latter statement gains weight when we come to think that aside from the formula for the thigh muscles being the same in *Cypseli* and *Trochili*, their pelvic limbs otherwise are by no means alike in other particulars; and the sternum is, too, of a very different pattern in each case, although, as I say, each possesses an entire posterior xiphoidal margin.

My object will have been attained here when I have presented you with a brief description of each of these muscles, and directed your attention to them in my drawings which illustrate this paper. As will be seen by the figures, I found them *all* present in the pelvic limb of *Geococcyx californianus*, and this is the bird I have chosen to illustrate my remarks in what follows.

6. The *ambiens* (Fig. 9): This muscle arises from the apex of the prominent prepubic spine of the pelvis, and the fibres passing directly down to the inner side of the femur, and parallel with that bone, form a strong fusiform muscle. As it approaches the patella it terminates in a small flattened tendon, which, piercing the fascial envelop of the knee-joint below the inferior apex of that sesamoid, passes round the joint to become finally lost to the outer side and opposite the summit of the tibia, where some of its tendinous fibres merge with the fibres of origin of the

*flexor perforatus digitorum*, or, at least, with one of its divisions.

The *ambiens* is overlaid by the *sartorius* muscle, and in the figure is brought into view only through the aid of a small dissecting-hook and chain, which pull it forwards in order that it may be better seen.

7. The *femoro-caudal* (Fig. 9) arises, tendinous, from the lower posterior border of the pygostyle. It soon becomes fleshy and as a narrow, muscular ribbon passes through the tissues overlying the lateral group of caudal muscles proper. Opposite the posterior border of the pelvis it expands to form a prettily shaped and compressed spindle, closely covering the *obturator externus* muscle and the side of that bone. As it nears the femur it again contracts, receives the fibres of its accessory head, and is finally inserted upon the femoral shaft, at the posterior aspect of its proximinal third.

8. The *accessory femoro-caudal* arises beneath the overarching part of the postacetabular portion of the ilium, just behind the acetabulum and beyond. Its fibres pass obliquely downwards and forwards to join with those of the *femoro-caudal*, and to become inserted with them into the upper part of the femur as already described.

9. The *semitendinosus* (Figs. 8 and 9) is a marvelously well-developed muscle in this form, as is also its accessory head. Its origin fills about three-fourths of the nether cavity formed by the posterior over-arching portion of the ilium, under which it arises.

Posteriorly, the fibres forming its free margin are so arranged as to create a rounded border ; the lower end of its arc terminating about opposite the post-pubis of the pelvis. From this origin the fibres of the *semitendinosus* pass downwards and forwards as a great, though somewhat compressed muscle. When within rather more than a centimetre's length of the shaft of the femur, they terminate in an oblique tendinous raphe, which latter forms the bounding-line between this muscle and the next.

10. The *accessory semitendinosus* (Figs. 8 and 9), is composed of coarser fibres than the muscle just described. It

Fig. 9. Pelvic limb, right side, of *G. californianus*, same specimen as is shown in figures 7 and 8. Still other muscles have now been removed, and the *ambiens* is in full view, while other interesting parts are brought into sight. Life size by the author from his own dissections.

springs from a longitudinal line occupying the distal half of the shaft of the femur, and from the upper surface of the hinder aspect of the external condyle of that bone. The fibres pass backwards and a little upwards to become inserted into the tendinous raphe just alluded to. The lower extremity of this tendinous raphe terminates, in *Geococcyx*, in a thin, flat, and delicate tendon, which continues downwards and forwards to the inner surface of the head of the tibia, where it becomes inserted, the point of insertion being found above that of the *semimembranosus* muscle, the insertional tendon of which overlaps it.

As in the case with the other muscles described in the foregoing paragraphs, ornithotomists have a fine field open before them in dissecting out this group of thigh muscles in our United States birds; making full notes upon their researches, and comparing carefully with the work already accomplished by the indefatigable Garrod. In doing this, not merely the absence or presence of the five muscles last described should be noted, but if possible, full notes made as to their *exact* origins and insertions, their *relative* size as compared with other allied birds, and in short their morphology in its details.

### THE AREA OF THE ORIGIN OF THE OBTURATOR INTERNUS.

In birds this muscle arises, as shown in the figure, from the central surface of the pelvis, its fibres being attached to the post-pubic bone and the ischium. As a rule it is a bipenniform muscle, its fibres being directed forwards, but at the same time, on either side of its own moiety, towards a longitudinal tendinous and mid-line of its own. This tendon becomes stronger as it approaches the obturator foramen, and passing through this, is finally inserted into the head of the femur of the corresponding side, and upon its outer surface.

Now in a great many birds the area from which the *obturator internus* arises is of an *oval* outline, while on the other hand in nearly an equal number of the class, this area will be found to be a *triangular* figure. So it has been said, that it can thus be utilized as a good character, in this way, when taken in connection with others. In some few birds I understand, it is difficult to determine whether this area of origin is *oval* or *triangular*, but as a rule no such difficulty presents itself. For my own part it constitutes a difference which, I am free to confess, I had as yet paid but little attention to, as for several years past neither the proper material nor other facilities for such investigations have been available. Nor am I quite sure in my own mind as yet, how far the form of the hinder portion of the pelvis may influence the origin of this muscle; and whether such birds do not exist wherein a large *obturator internus* is demanded, and where their pelves are *short*, in which cases the muscle, to gain a firmer origin, would naturally spread out posteriorly, and thus of necessity become *triangular*.

But as I say, I am not prepared to pass final judgment on this matter, and render a personal opinion as to whether much reliance can be placed upon it as a useful character in determining affinities among birds.

Here then again is a field open to decide an important point, and one easily to be understood, and not difficult to render extensive records about. Those living where land and water birds occur in abundance could soon determine whether (or no) this character possessed any taxonomic value or not, and the result would surely prove of service to ornithology.

### ON THE MUSCULATURE OF THE SYRINX.

Research of a far more extensive character will have to be made in this direction before results can be attained, and data secured which can be generally used with effect in classification. Much of course is already known, as we are aware for instance, that in the true oscine type four or five pairs of intrinsic muscles are found associated with the syrinx, and these are inserted into the three upper bronchial semi-rings *at their extremities*; hence these birds have been designated as the Passeres Acromyodi, and in them the syringeal apparatus is of a very complex nature, holding the highest place among birds as an organ of voice. On the other hand we have the Passeres Mesomyodi, or the

Fig. 10.—Pelvic limb, right side, of *Geococcyx californianus*, same specimen as is shown in figures 7, 8 and 9. The deepest muscles of all are now only left, and the tendon of insertion of the *obturator internus* is distinctly seen. Life size by the author from his own dissections.

Clamatorial Birds, wherein the intrinsic muscles of the syrinx are fewer than four pairs in number, and where they are inserted into the middle points of the upper semi-rings of the bronchus; and consequently the apparatus here is of a less complicated and of a less perfectly developed a character as an organ of voice. As examples of the first group we have the Thrushes, the Wrens, the Warblers, the Finches, the Crows and many others; and in the second group the Tyrant Flycatchers.

Where some good work remains to be done, however, among the birds in this country, is in the various groups of the so-called water birds; in the Gallinæ and in the Picidæ, and other groups. The form the trachea itself assumes is, of course, of prime importance in these birds, and in many cases the muscular part of the apparatus will be found to be but feebly developed.

### ON THE ARRANGEMENT OF THE DEEP PLANTAR TENDONS IN BIRDS.

Both Professor C. J. Sundervall and Professor Garrod have paid considerable attention to the disposition of these tendons in the feet of birds. If I mistake not, the first-named author was the writer who originally invited attention to the fact that the tendon of the *flexor longus hallucis* was completely independent of the tendon of the *flexor perforans digitorum* in the Passeres; and in view of this fact he grouped these birds together and as the Hoopoe (*Upupa*) exhibited the same condition, he included that form with them. Garrod pushed the matter much further, however, and made some very extensive dissections upon the deep plantar tendons in a great many different orders of birds.

Irrespective of the plan of the foot, in all birds, in so far as its digits are concerned, there are two muscles present in the leg, which, arising from the tibia and fibula, send each a tendon to the toes as *flexors*, these muscles are the *flexor longus hallucis* and the *flexor perforans digitorum pedis.* In passing through or over the hypotarsus of the tarso-metatarsus, at the back of the ankle-joint, the tendon of the *flexor longus hallucis* is either *superficial* or *external* to the tendon of the other flexor mentioned.

This fact is useful to be borne in mind in identifying these tendons in our dissections. Now after they pass a short distance down the back of the tarso-metatarsal bone their behavior in different birds is quite diverse, and a few examples of it will be here presented in order to show that when our knowledge becomes more full in the premises, the character will prove a useful one in classification of birds; and also it is hoped to induce those interested in the science of anatomy to undertake and carefully record researches upon this subject. Before arriving at the podal phalanges, and in the sole of the foot of any bird, these tendons divide into a sufficient number of slips to be distributed to the former, one slip going to each toe. The method of division is the same for the bulk of avian families, and the more universal type is well exemplified in the Common Chicken (*Gallus*).

Figure 12 of this paper shows this arrangement in the Fowl, and there we observe that the tendon of the flexor to the first toe is *external* to the tendon of the *flexor perforans digitorum* as it passes the ankle-joint.

At the back of the tarsus it crosses the latter superficially, and then passing directly to the under side of the hind-toe it becomes inserted into the base of its ungual phalanx.

Now the larger tendon of the *f. p. digitorum*, after arriving at the sole of the foot, trifurcates, and a slip is sent to the under side of each anterior toe, where passing forwards they too become inserted at the bases of the ungual digits of the respective phalanges. Just above this trifurcation the tendons of these two muscles, however, are connected, and that by a fibrous *vinculum* (Fig. 12, V) which passes between them.

The fibres of this vinculum come off from the tendon of the *flexor longus hallucis*, and pass downwards to soon merge with the fibres of the tendon of the *flexor perforans digitorum* at the crossing. This arrangement, of course, influences the action of flexion in the foot of the passerine bird, but this question does not especially concern us here.

The strength and size of the vinculum is different in nearly every group of birds where it is present. In figure 10 at *a*, I have shown the position and size of this vinculum as I found it to be present in the Ground Cuckoo.

Fig. 11

Fig. 12.

Fig. 13.

Fig. 11. Right foot of *Megalœma asiatica*, showing an arrangement of the plantar tendons.

Fig. 12, Left foot of *Gallus bankiva*. V. vinculum running downwards from the outer hallucial tendon to the inner digital common tendon.

Fig. 13. Right foot of *Apteryx mantelli*. (All three figures copied by the author from Garrod.)

In order to show how different this disposition of these plantar tendons may be, I quote Garrod's description of his dissection of the foot of a specimen of *Megalœma asiatica* (fig. 11). He says, in this bird "the two tendons descend behind the ankle as usual, having their origins typical. There is nothing peculiar till they have descended two thirds down the tarso-metatarse. About opposite the middle of that bone the *flexor longus hallucis* sends a vinculum downwards as in the Fowl, to join the tendon of the *flexor perforans digitorum.* Just above the metatarso-phalangeal articulation the tendons become arranged for distribution in a most uncommon manner. The tendon of the *flexor perforans digitorum* does not split up, but runs to one digit only, namely, the third toe, which is the outer of the two that are directed forward. It is covered superficially by the *flexor perforans digitorum*, just as that latter muscle is splitting up to be distributed to the hallux as well as to digits 2 and 4. In these birds we have, therefore, the *flexor longus hallucis* arising from the lower surface of the femur only, running through the ankle at the outer side of the other deep tendon, and sending a vinculum downwards—all of which are special characters of that muscle only, it being distributed to three toes, whilst the *flexor perforans digitorum* only supplies one."

In figure 13 the arrangement in the *Apteryx* is well shown, where the vinculum is so large as compared with this band in the Fowl, that it almost appears upon sight that the two flexor tendons are fused into one common one, at the point of union.

A close study of this arrangement, however, will throw much light upon the variations as they are to be found in the less typical birds in this respect.

## Figures and Tables

**Fig. 1. f1:**
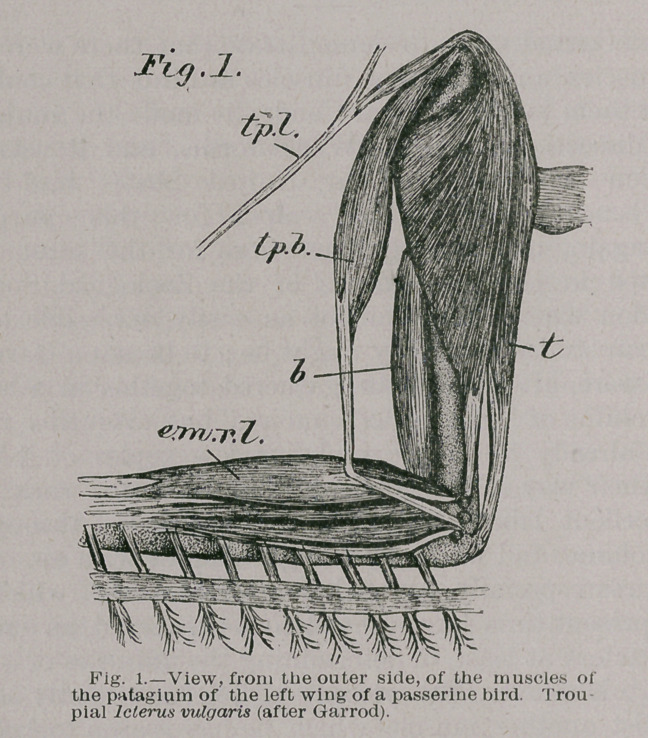


**Fig. 2. f2:**
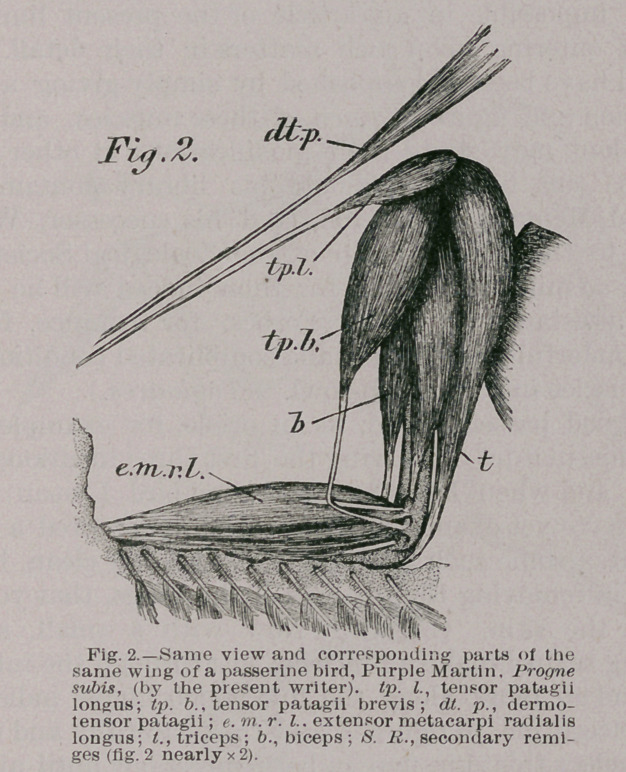


**Fig. 3. f3:**
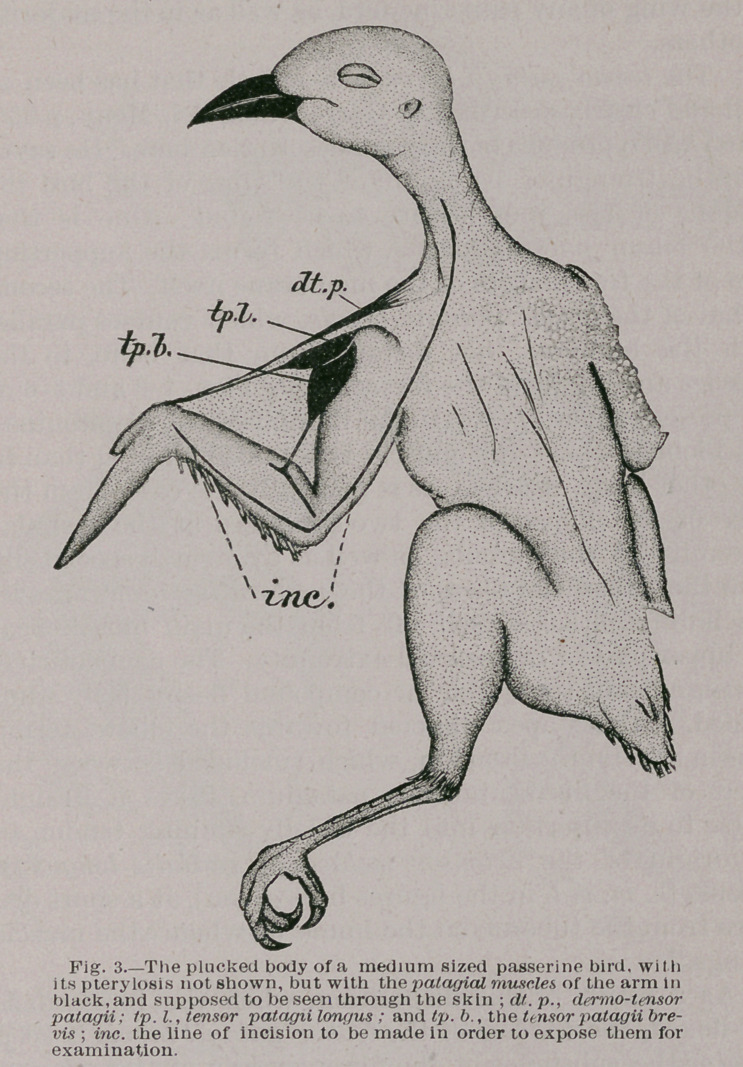


**Fig. 4. Fig. 5. Fig. 6. f4:**
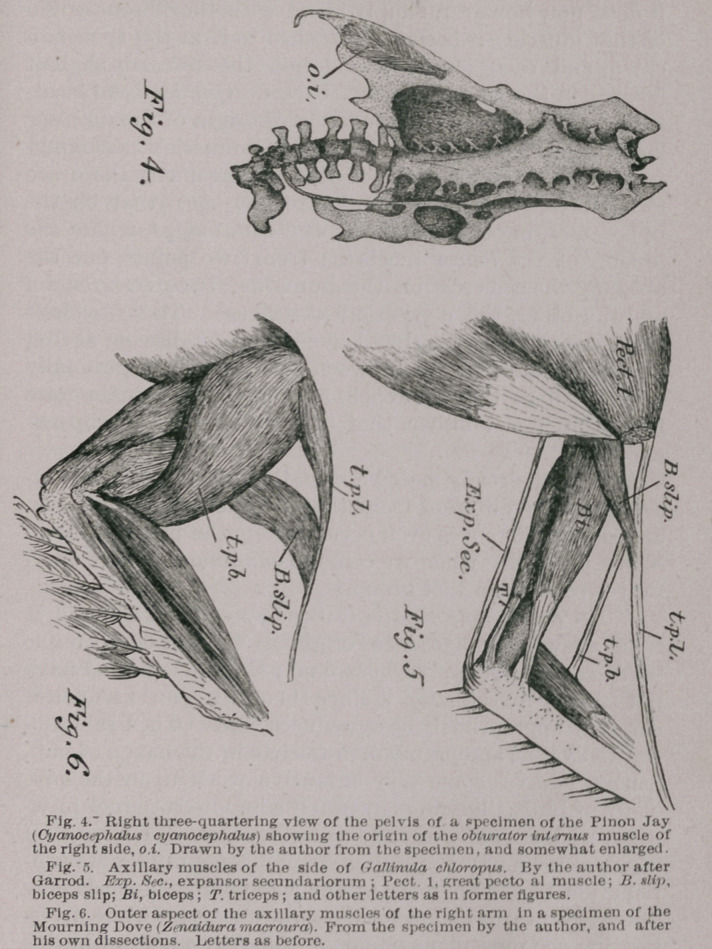


**Fig. 7. f5:**
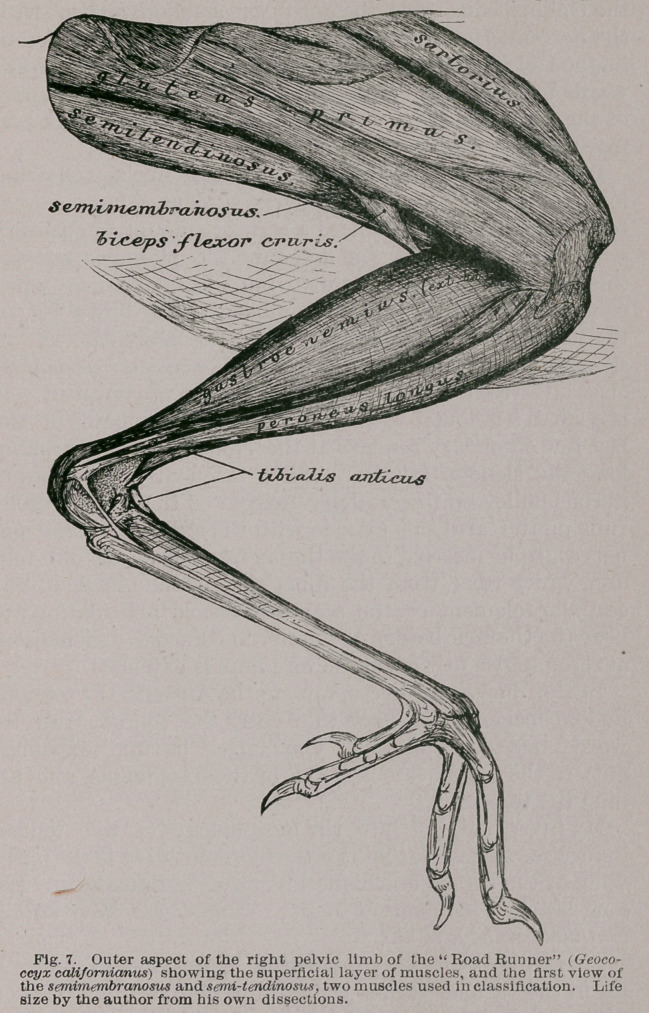


**Fig. 8. f6:**
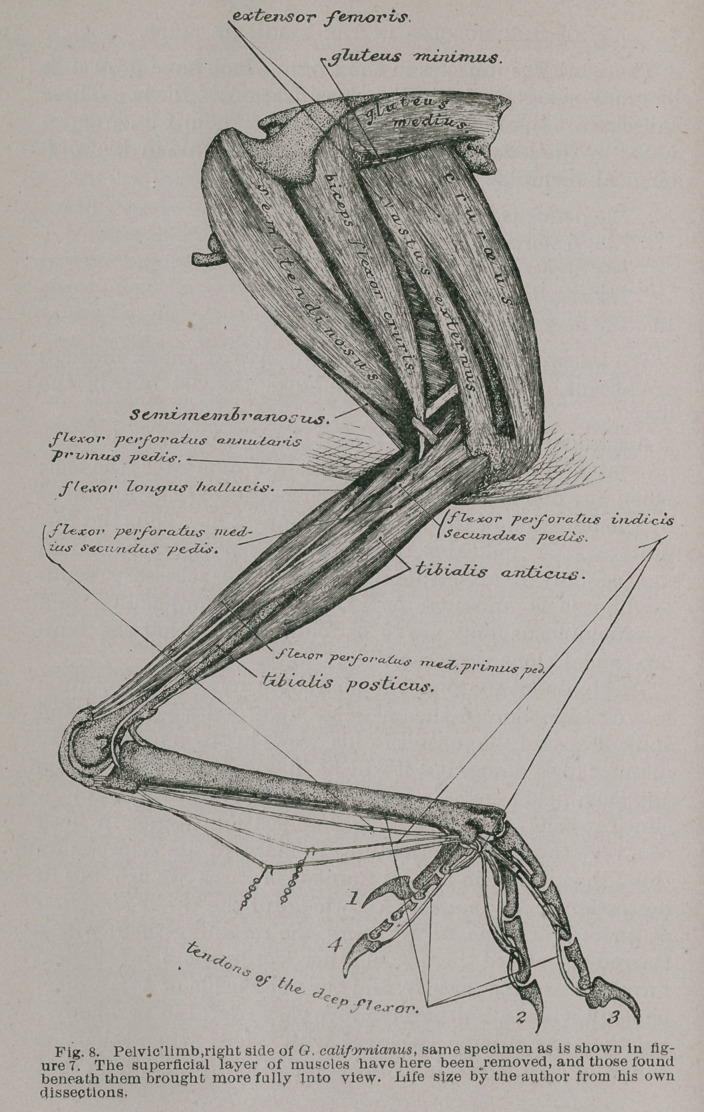


**Fig. 9. f7:**
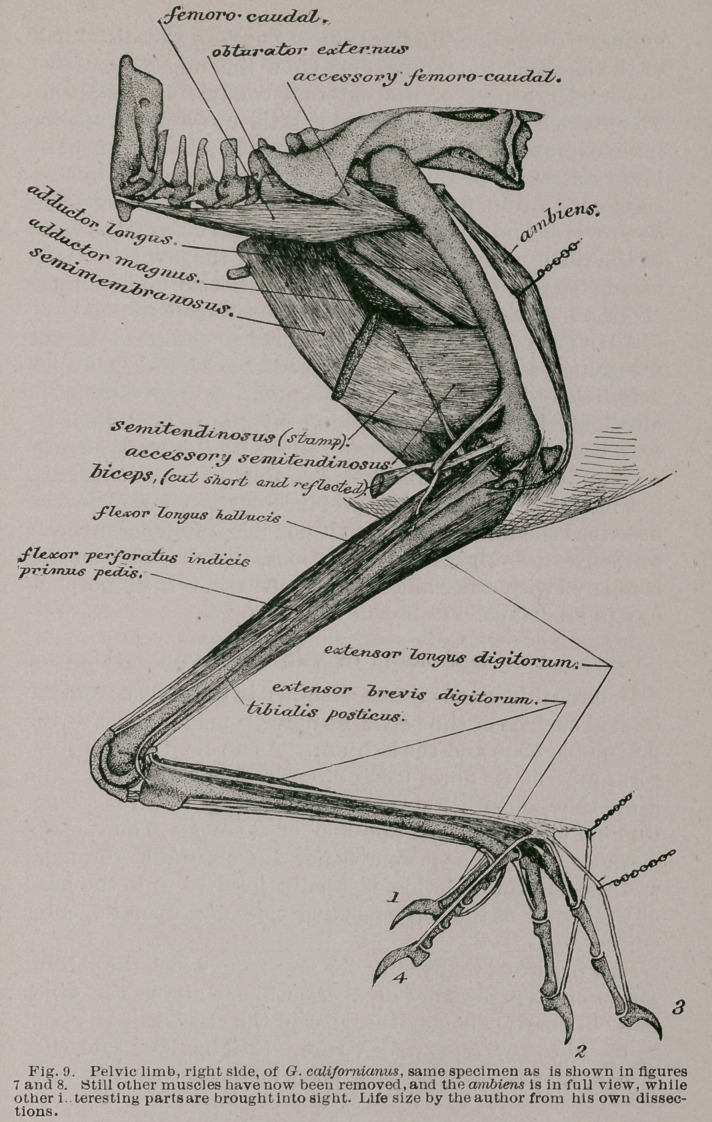


**Fig. 10. f8:**
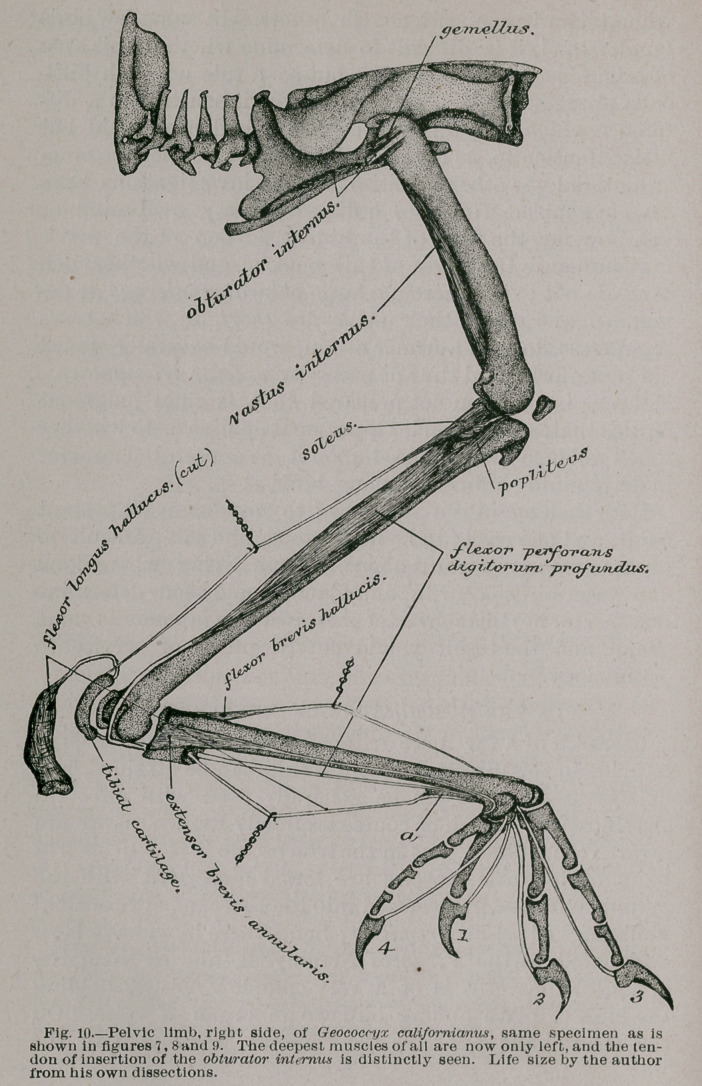


**Fig. 11. Fig. 12. Fig. 13. f9:**